# SWI/SNF chromatin remodeling complex and glucose metabolism are deregulated in advanced bladder cancer

**DOI:** 10.1002/iub.2254

**Published:** 2020-02-19

**Authors:** Malgorzata Stachowiak, Michal Szymanski, Anna Ornoch, Iga Jancewicz, Natalia Rusetska, Alicja Chrzan, Tomasz Demkow, Janusz A. Siedlecki, Tomasz J. Sarnowski, Elzbieta Sarnowska

**Affiliations:** ^1^ Department of Molecular and Translational Oncology Maria Sklodowska‐Curie National Research Institute of Oncology Warsaw Poland; ^2^ Department of Uro‐oncology Maria Sklodowska‐Curie National Research Institute of Oncology Warsaw Poland; ^3^ Department of Pathology Maria Sklodowska‐Curie National Research Institute of Oncology Warsaw Poland; ^4^ Institute of Biochemistry and Biophysics Polish Academy of Sciences Warsaw Poland

**Keywords:** AMPK, bladder cancer, chromatin remodeling, glucose metabolism, SWI/SNF complex

## Abstract

Bladder cancer (BC) is a frequently diagnosed malignancy affecting predominantly adult and elderly populations. It is expected that due to the longer life time, BC will become even more frequent in the future; thus in consequence, it will represent serious health problem of older society part. The treatment of advanced BC is mostly ineffective due to its very aggressive behavior. So far, no effective targeted therapy is used for BC treatment. Here, we found that BC is characterized by lower protein levels of BRM, INI1, and BAF155 main subunits of SWI/SNF chromatin remodeling complex (CRC) which is involved in global control of gene expression and influences various important cellular processes like: cell cycle control, apoptosis, DNA repair, etc. Moreover, the expression of *SMARCA2*, a BRM encoding gene, strongly correlated with BC metastasis and expression of such metabolic genes as *PKM2* and *PRKAA1*. Furthermore, the analysis of T24 and 5637 commonly used BC cell lines revealed different expression levels of metabolic genes including *FBP1* gene encoding Frutose‐1,6‐Bisphosphatase, an enzyme controlling glycolysis flux and gluconeogenesis. The tested BC cell lines exhibited various molecular and metabolic alterations as well as differential glucose uptake, growth rate, and migration potential. We have shown that BRM subunit is involved in the transcriptional control of genes encoding metabolic enzymes. Moreover, we found that the *FBP1* expression level and the SWI/SNF CRCs may serve as markers of molecular subtypes of BC. Collectively, this study may provide a new knowledge about the molecular and metabolic BC subtypes which likely will be of high importance for the clinic in the future.

**Abbreviations**
BC: bladder cancer
MIBC: muscle invasive bladder cancer
PKM: pyruvate kinase M
PRKAA1: AMPKα1 encoding gene
SWI/SNF: ATP‐dependent chromatin remodeling complex
TCGA: The Cancer Genome Atlas

## 1 INTRODUCTION

Bladder cancer (BC) is the one of the most commonly diagnosed malignant cancers all over the world. This disease is reported as the 13th cause of death.^1^ In western countries, the muscle invasive BC (MIBC) represents around 25% of all newly diagnosed BC cases. Up to 30% of non‐muscle invasive BC (NMIBC) develops progression to muscle invasive BC.^2^ Additionally, in 5–15% of BC patients the unresectable or metastatic disease is found at the time of diagnosis.^3^ Molecularly and clinically, BC is a heterogeneous disease. The large scale transcriptomic analysis revealed the existence of the luminal and basal subtypes of MIBC, similarly as it is reported for breast cancer.^4^ The basal subtype had much worse prognosis and survival rate than luminal subtype.^5^, ^6^ So far, no targeted therapy is recommended to treat advanced and/or metastatic BC and palliative cystectomy is performed. The prognosis in metastatic BC is generally extremely poor.^7^

Mutations in various genes were identified in BC. One of them is *ARID1A* truncating mutation^8^ affecting the production of proper BAF250a subunit of SWI/SNF ATP‐dependent chromatin remodeling complex (CRC).^9^ The SWI/SNF CRC is composed of several core and non‐core subunits. The core complex of canonical SWI/SNF CRCs is formed by one ATPase subunit: BRM or BRG1; two SWI3‐type proteins: BAF155 and/or BAF170 and one SNF5‐type INI1 protein.^9^ Subunits of SWI/SNF CRC were found to be dysregulated in ccRCC and other malignant cancers.^10^, ^11^, ^12^, ^13^, ^14^ Noteworthy, the loss of some SWI/SNF subunits was observed in undifferentiated/dedifferentiated urothelial cancers, mostly BCs.^15^

In this study we found that BC is characterized by downregulation of BRM, BAF155, and INI1 the core subunits of SWI/SNF CRCs in advanced BC samples derived from 45 patients. The expression of *SMARCA2*, a BRM ATPase encoding gene, strongly correlated with the expression of *PKM2* and *PRKAA1* genes, and metastasis, indicative of the existence of metabolic alterations in BC. We found that the T24 and 5637 BC cell lines are featured by differential expression of genes coding for crucial metabolic enzymes, various abilities to glucose uptake, and different aggressiveness. The differential characteristics of T24 and 5637 cell lines including the differential expression of EMT marker genes suggests that they may represent various BC subtypes. The observed alterations of various metabolic enzymes indicate that impaired glycolysis flux and gluconeogenesis may be characteristic features of the BC basal subtype. Subsequently, we have shown that the *FBP1* expression level and the SWI/SNF CRCs may serve as signatures for molecular subtypes of BC.

## 2 EXPERIMENTAL PROCEDURES

### 2.1 Ethics statement

This study was performed in accordance with the Declaration of Helsinki and upon approval by the Ethics Committee in Maria Sklodowska‐Curie National Research Institute of Oncology (MSCNRIO), Warsaw, Poland. All patients were informed about study and provided written informed consent, and were informed that their privacy would be maintained.

### 2.2 Study subjects

Biological material was obtained from Maria Sklodowska‐Curie National Research Institute of Oncology in Warsaw. Forty‐five formalin‐fixed, paraffin‐embedded samples of patients with BC were analyzed. All samples were characterized by pathologist for type, stage (Tumor, Node, Metastasis [TNM] classification) and tumor grade.

### 2.3 Immunohistochemistry staining and scoring

Immunohistochemistry (IHC) was performed on 3.5‐μm sections of paraffin‐embedded tissue samples. These sections were deparaffinized with xylene and rehydrated with descending ethanol concentrations (from 96 to 50%). Antigen exposure was obtained by heat‐induced epitope retrieval performed in Target Retrieval Solution (Dako) for 25 min at 96°C. After cooling, tissues were treated with Peroxidase Blocking Reagent (Dako) for 5 min, followed by incubation with appropriate monoclonal antibody (Supplementary Table 1) and labeled with EnVision FLEX+, Mouse, High pH Detection System (Dako). The color product occurred as a result of horseradish peroxidase and 3,3′‐diaminobenzidine tetrahydrochloride reaction. Nuclear imaging was visualized with hematoxylin staining (DAKO) for 20 s. Histological score (H‐score) was calculated according to the following formula:

H−score=3*percentage of high stained cells+2*percentage of medium stained cells+percentagelowstained cells

### 2.4 Cell culture and transfection

T24 (Human urinary bladder with transitional cell carcinoma) and 5637 (Human urinary bladder grade II carcinoma) cell lines were obtained from ATCC global biological resource and standards organization. All lines were authenticated by ATCC. T24 cells were cultured in DMEM (Biowest) supplemented with 10% FBS (Biowest) and 5637 cells—in RPMI (Biowest) with 10% FBS (Biowest). Culture conditions were 37°C, 5% CO_2_, and 95% humidity. These adherent cell lines were passaged once in 3–4 days by treatment with trypsin (Biowest). For BRM transient overexpression the pcDNA6.2 vector and pcDNA6.2 with cloned in frame full length BRM cDNA (BRMox) were used. The transfection of the BC cell lines was performed using Lipofectamine 2000 according to the protocol. Transfection level was evaluated using qRT‐PCR with *SMARCA2* specific primers.

### 2.5 qRT‐PCR

Total RNA was isolated from ~5 × 10^6^ of T24 and 5637 cells using ReliaPrep RNA Miniprep Systems (Promega) according to manufacturer's protocol. One microgram of total RNA was transcribed by reverse transcriptase using Transcriptor First Strand cDNA Synthesis Kit (Roche) according to the standard protocol. Gene expression was evaluated using the comparative CT (2^−ΔΔCT^) method with SybrGreen (BioRad) and ubiquitin as the reference gene. Primers' sequences specific for studied genes are present in Supplementary Table 2.

### 2.6 Western blot

Total protein extract was isolated from 3 × 10^6^ of T24 and 5637 cell lines pellets. Thirty micrograms of protein extract was loaded on each line of 12% SDS‐PAGE gel with 0.5% TCE (BioRad). After separation, the proteins were visualized on UV light, in order to examine proper protein loading (stain‐free loading control).^16^

Western blotting using in‐gel protein labeling as a normalization control: stain‐free technology^16^ and transferred (70 V, 2 h, and 4°C) on PVDF membrane (Milipore). Membrane was blocked on 5% milk in TBS with 0.1% Tween‐20 (TBS‐T). Incubation with primary antibodies was performed at 4°C, overnight. Antibodies details and working concentrations are present in Supplementary Table 3. After incubation with primary antibodies, membranes were washed three times for 5 min in TBS‐T and probed with secondary antibodies (Supplementary Table 3) for 1 hr at RT. Chemiluminescence signal was detected by WesternBright Quantum detection kit (Advansta) using Azure Biosystems chemiluminescence detection system.

### 2.7 Transcriptome database analysis

The microarray datasets obtained from Gene Omnibus Database (GEO) with accession number GSE31684 from 93 BC samples^17^, ^18^ were reanalyzed using GeneSpring GX software (Agilent) according to the advanced workflow.

### 2.8 Statistical analysis

Statistical analysis was performed using GraphPad Prism 5.0 software by Shapiro–Wilk normality test, paired *t*‐test, Mann–Whitney rank test for independent samples and multiple regression. *p*‐value < .05 was considered as statistically significant. Log‐rank test was used to estimate probability of surviving. The relationship between *SMARCA2* expression and expression of other genes was estimated using Pearson correlation coefficient.

### 2.9 SurvExpress databases

In this study the survival analysis was performed for *SMARCA2* and *FBP1* genes on BC data‐based deposited in GEO accession number GSE13507^19^ using the SurvExpress bioinformatics tool (http://bioinformatica.mty.itesm.mx:8080/Biomatec/SurvivaX.jsp).^20^

### 2.10 Ualcan database

Ualcan (http://ualcan.path.uab.edu) is an online available tool, which uses The Cancer Genome Atlas (TCGA) RNA‐seq and clinical data from various cancer types. In this study, Ualcan was used to compare the expression of *SMARCA2*, *SMARCC1*, and *SMARCB1* in TCGA BC samples and for comparative analysis of tumor and normal tissue.^21^ The 550 genes positively correlated with *SMARCA2* expression were further used for Gene Ontology analysis using GO tools https://go.princeton.edu/. The GO analysis was performed using GO Term Finder tool, processes as ontology aspect and *H. sapiens* as annotation.

### 2.11 Glucose uptake analysis

Glucose uptake by the T24 and 5637 cell lines was measured using Glucose Uptake‐Glo™ Assay (Promega). Five thousand cells of each line were plated into 96‐well plate (0.32 cm^2^) and cultured for 24 hr and treated according to protocol.

### 2.12 Immunocytochemistry

Immunocytochemistry assay was performed on 40,000 cells plated into 4‐well plate LabTek® II Chamber Slide (Nunc). The cells were cross‐linked using 4% paraformaldehyde for 20 min and incubated with Triton X‐100 (Sigma‐Aldrich) for 5 min. Endogenous signal was blocked using 1% BSA (Bioshop) solution (in PBS) for 30 min. The following steps, starting from specific antibody incubation (Supplementary Table 1), were performed as it was described in Immunohistochemistry subsection.

### 2.13 Cell growth analysis

Ten thousand cells of T24 and 5637 cell lines were plated into 24‐well plate (1,9 cm^2^ each well) and cultured in appropriate conditions. Cells were counted using Neubauer hematocytometer (Sigma‐Aldrich) after every 24 hr for 5 days.

### 2.14 Scratch assay—Migration test

Five lakh cells of T24 and 5637 cell lines were plated into 6‐well plate (9.5 cm^2^ each well) and cultured in appropriate conditions for 24 hr, when the confluency of the cells achieved 100%. Then, the media were changed and supplemented with 60 μM hydroxyurea (Sigma‐Aldrich). Two hundred microliters pipette tips were used to obtain gaps through the center of well. Width of the gaps was captured and measured in respective time points: 0, 2, 4, 6, and 24 hr.

## 3 RESULTS

### 3.1 Characteristics of patients enrolled in this study

Forty‐five patients with diagnosed BC which underwent the cystectomy in MSCNRIO in Uro‐oncology Clinic are summarized in Table 1.

**Table 1:** Clinico‐pathological characteristics of patients enrolled in the study

Patients	Total number (*n* = 45)
Gender	
Female	8
Male	37
Years (mean)	
Female	67
Male	67
TNM classification	
pT1	4
pT2	7
pT3	18
pT4	16
Grade	
G0	‐
G1	‐
G2	‐
G3	45

Abbreviation: TNM, Tumor, Node, Metastasis.

### 3.2 Alterations in SWI/SNF core subunits abundance characterize advanced BC

**Figure 1:**
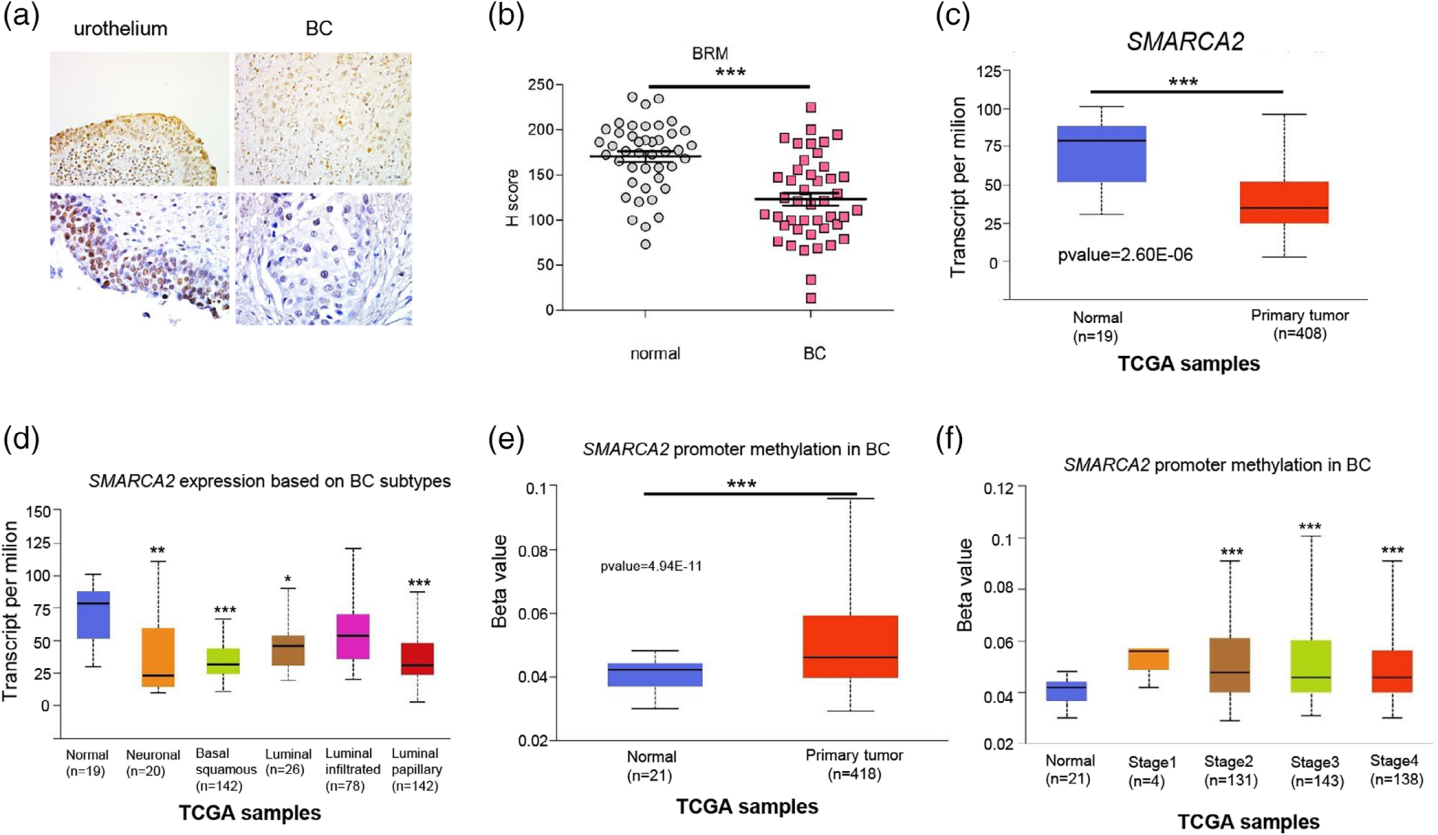
Downregulation of BRM, the central ATPase of SWI/SNF chromatin remodeling complex and *SMARCA2*, a BRM encoding gene, characterize bladder cancer. The protein level of BRM ATPase is decreased in advanced bladder cancer samples compared with normal urothelium—the IHC with anti‐BRM antibody (magnification 20× and 63×) (a). The statistical analysis of BRM abundance in 45 MIBC patient samples versus healthy tissue analyzed using H‐score method indicates significant BRM downregulation in BC, *p*‐value < .0001 (b). *SMARCA2*, a BRM encoding gene, exhibits downregulated expression BC samples versus normal tissue. The bladder cancer TCGA datasets were analyzed, *p*‐value = 2.60E‐06 (c). Different *SMARCA2* expression patterns are exhibited by various BC molecular subtypes: neuronal, *p*‐value = 4.22E‐04; basal squamous, *p*‐value = 5.70E‐10; luminal, *p*‐value = 1.33E‐02, luminal infiltrated, *p*‐value = 1.08E‐01; luminal papillary, *p*‐value = 4.49E‐08 (d). High methylation rate of *SMARCA2* gene promoter region in BC samples is observed when compared with healthy urothelium, *p*‐value = 4,92E‐11 (e). *SMARCA2* methylation status depends on stage of the disease: stage 1, *p*‐value = 1.75E‐01; stage 2, *p*‐value = 9.02E‐09; stage 3, *p*‐value = 9.53E‐09; stage 4, *p*‐value = 1.30–08 (f). The Beta value on the graph indicates level of DNA methylation 0—unmethylated and 1—fully methylated. BC, bladder cancer; IHC, Immunohistochemistry; TCGA, The Cancer Genome Atlas

The mutations in *ARID1A* gene encoding BAF250a subunit of the SWI/SNF CRC were found in BC, although mutations in genes coding for core subunits were identified only with low frequency.^8^, ^22^ The self‐regulation and interdependence between SWI/SNF subunits expression were already described^10^; therefore, we examined the abundance of three main SWI/SNF CRC subunits: BRM ATPase, BAF155, and INI1 in advanced MIBC compared with adjacent urothelium using IHC on paraffin‐embedded samples. The BRM protein was found in the nucleus in both normal and cancer cells (Figure 1a), but in BC the amount of BRM was significantly lower than in normal urothelium *p*‐value < .0001 (Figure 1b). The decrease of BRM abundance was observed in nearly all examined paired samples. Moreover, the TCGA dataset analysis revealed significant downregulation of *SMARCA2* (a BRM encoding gene) expression in BC compared with healthy tissue (Figure 1c). Of note, the strongest decrease of *SMARCA2* expression was observed in basal/squamous (*p*‐value = 5.7E‐10) and luminal papillary (*p*‐value = 4.48E‐08) molecular BC subtypes (Figure 1d). Consistently, the increased methylation of *SMARCA2* promoter region was observed in BC samples compared with normal tissue and in higher stage of the disease (Figure 1e,f).

**Figure 2:**
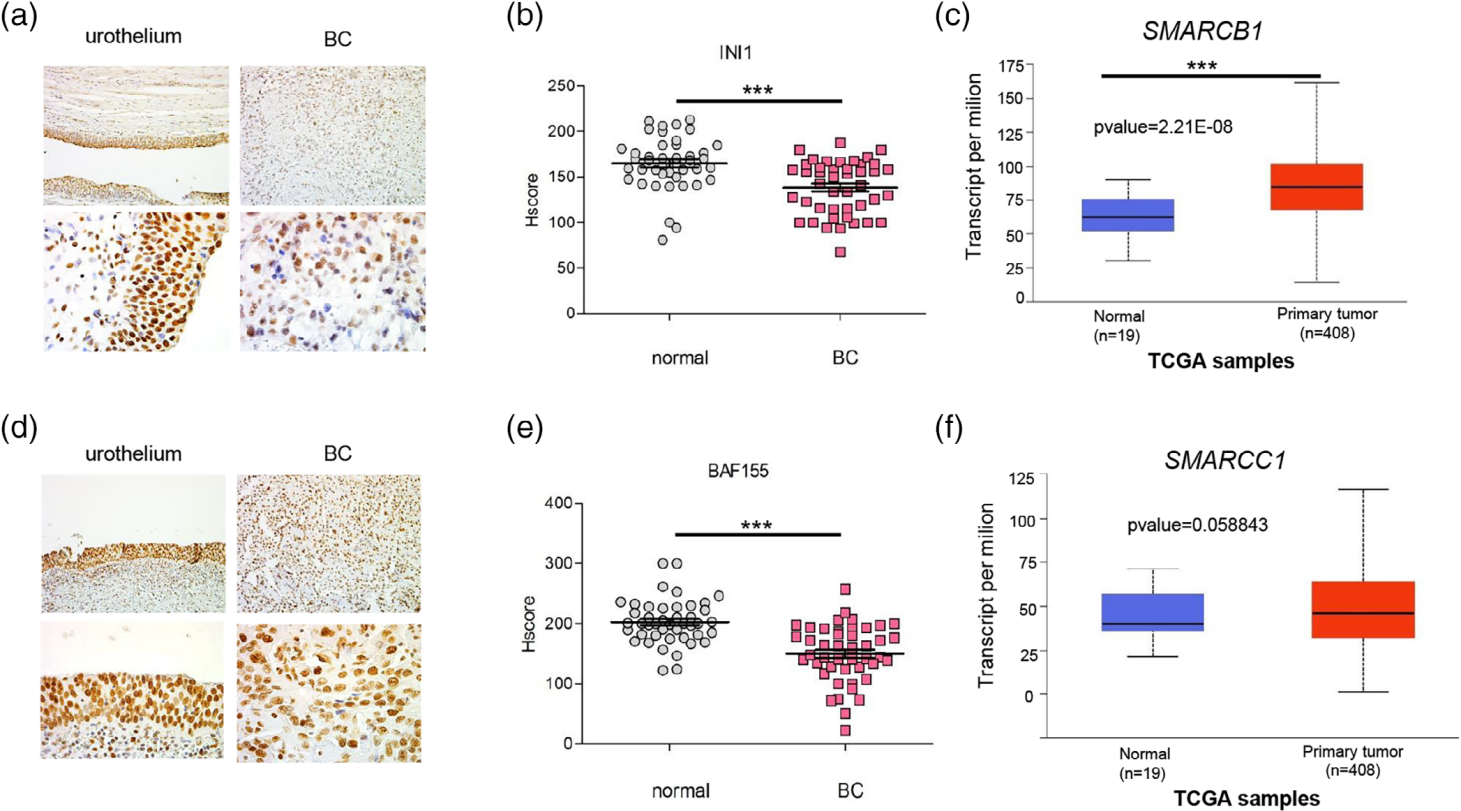
Bladder cancer is featured by downregulation of BAF155 and INI1 core subunits of SWI/SNF chromatin remodeling complex. Comparison of INI1 expression pattern in healthy urothelium and advanced bladder cancer tissue using IHC method, magnification 20× and 63× (a). INI1 abundance is decreased in MIBC samples when compared with normal tissue; the analysis was performed using H‐score calculation method, *p*‐value < .0001 (b). The primary BC tumor is characterized by the overexpression of *SMARCB1*, the INI1 encoding gene—TCGA BLCA datasets analysis, *p*‐value = 2.21E‐05 (c). MIBC is characterized by the statistically significant lower BAF155 protein level when compared with healthy urothelium; IHC using anti‐BAF155 antibody, magnification 20× and 63× (d). The BAF155 subunit of SWI/SNF CRCs is less abundant in BC when compared with normal tissue. The H‐score analysis of 45 patients cohort, *p*‐value < .0001 (e). The *SMARCC1* gene is expressed at similar level in primary BC tumor and normal tissue. TCGA BLCA datasets analysis *p*‐value = .0588 (f). BC, bladder cancer; CRC, chromatin remodeling complex; IHC, immunohistochemistry; TCGA, The Cancer Genome Atlas

The IHC examination demonstrated additional strongly decreased abundance of INI1 protein in BC (*p*‐value = .0005, Figure 2a,b) further indicating the aberrations of SWI/SNF CRCs in this type of cancer; however, in some cases, the INI1 level was not changed or even increased in cancer cells compared with the corresponding healthy urothelium. Surprisingly, the expression of *SMARCB1* gene encoding INI1 was increased in BC compared with healthy tissue (Figure 2c).

The BC cells were characterized by the significant decrease of BAF155 protein, *p*‐value < .0001 (Figure 2d,e); however, similarly as for INI1 protein in some cases, no differences between normal tissue and cancer were found. According to TCGA database, the expression levels of *SMARCC1* gene encoding BAF155 protein were similar in BC cells and healthy tissue (Figure 2f). No correlation between abundance of BRM, INI1, and BAF155 in BC cells was observed (data not shown). Collectively, our results show the deregulation of various SWI/SNF CRC classes in BC.

### 3.3 Low expression of BRM encoding gene associates with patient poor survival

**Figure 3:**
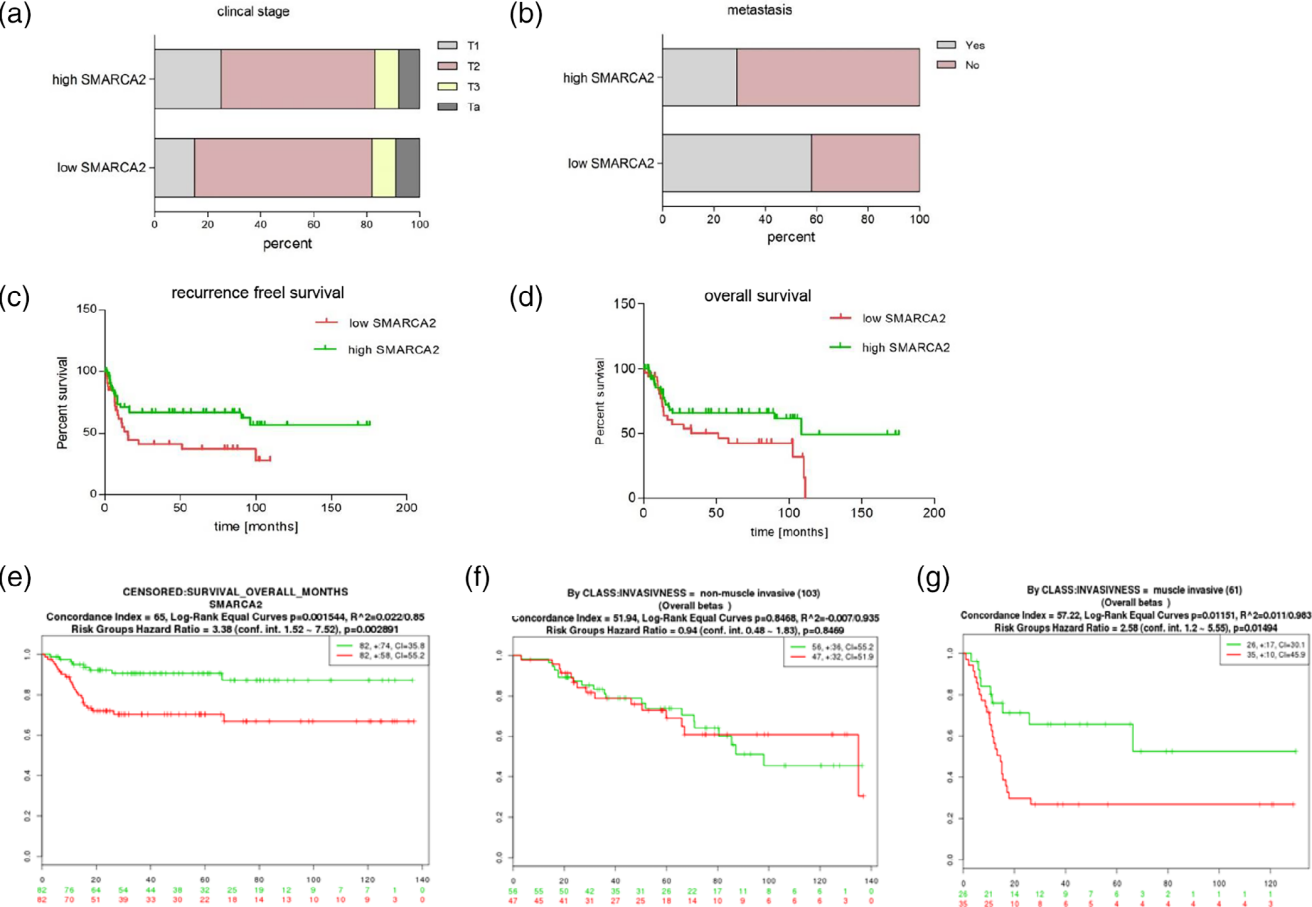
The altered expression of *SMARCA2* correlates with clinical observations. There is no correlation between low and high *SMARCA2* expression and clinical stage of the disease, *p*‐value = .36 (a). Low *SMARCA2* expression correlates with metastasis frequency, *p*‐value < .0001 (b). Low *SMARCA2* expression correlates with worse recurrence free survival, *p*‐value = .0217 (c). Worse overall survival of the patients, *p*‐value = .0397 (d). Low expression level of *SMARCA2* correlates with reduced survival rate for BC patients (e). There is no correlation between *SMARCA2* expression level and overall survival in MIBC patients (f). There is correlation between *SMARCA2* expression level and MIBC patients (g). BC, bladder cancer; MIBC, muscle invasive bladder cancer

We found that BRM protein level is downregulated in the most of analyzed BC samples, thus we decided to reanalyze microarray dataset available in Gene Expression Omnibus database (GEO accession number GSE31684^17^). This analysis revealed that the low *SMARCA2* gene expression did not correlate with clinical stage of the disease (Figure 3a) but we found a strong correlation between low *SMARCA2* transcript level and BC metastasis *p*‐value < .0001 (Figure 3b). The survival analysis using log‐rank test indicated the lower recurrence free survival for patients with low expression of *SMARCA2* gene (Figure 3c) *p*‐value = .0217 and the overall survival was significantly lower for BC patients with low expression of BRM encoding gene—*SMARCA2* (Figure 3d). To confirm the association of *SMARCA2* expression with BC patients' survival the analysis of large datasets (GSE13507)^19^ was performed using SurvExpress. Interestingly, the low *SMARCA2* expression was associated with poor survival of BC patients (Figure 3e), predominantly for patient with muscle invasive BC but not for NMIBC (Figure 3f,g).

### 3.4 SMARCA2 expression correlates with PKM isoform 2 (PKM2) and PRKAA1 gene expression in BC tumors

**Figure 4:**
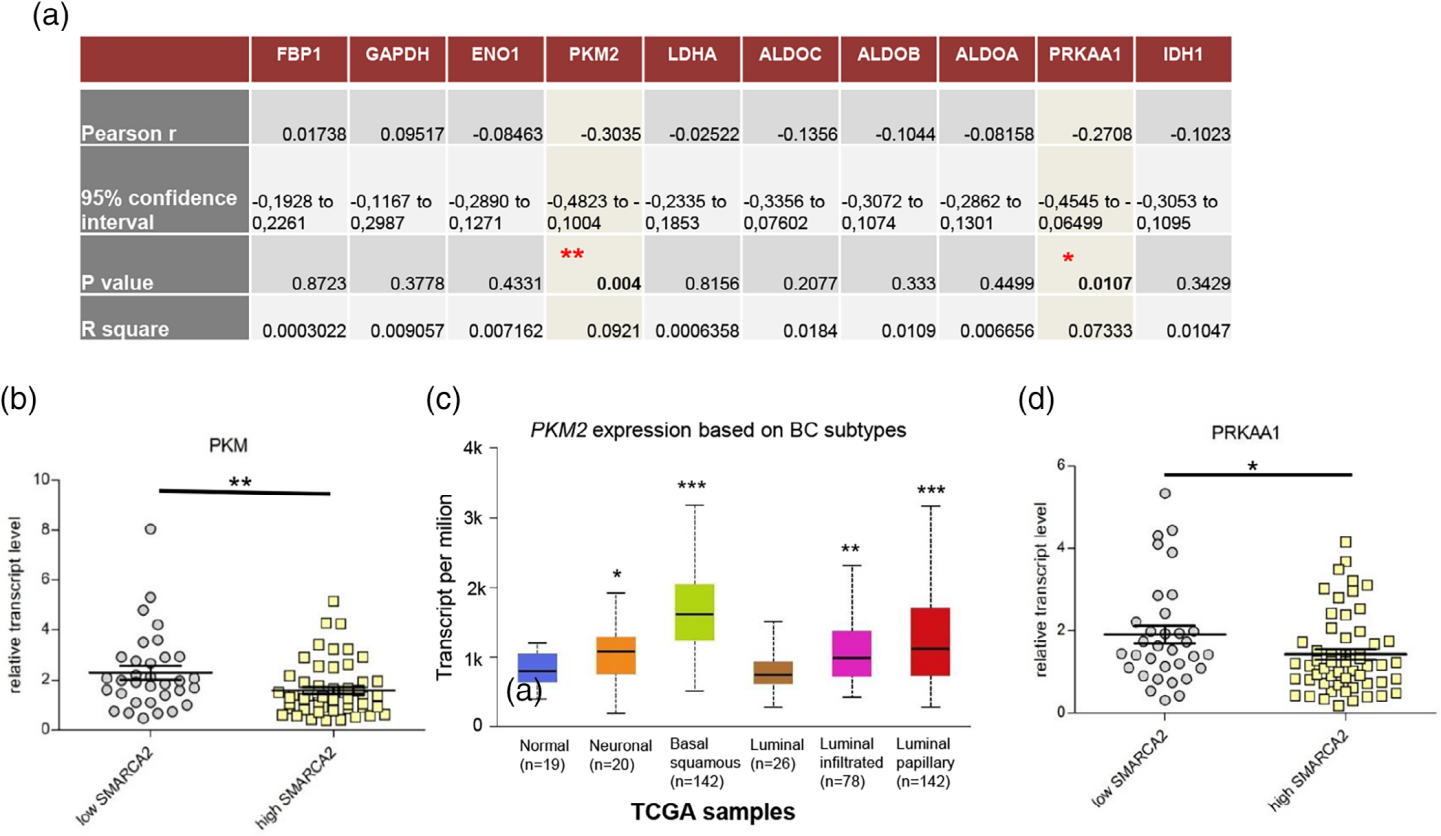
Metabolic alterations characteristic for bladder cancer correlate with patient outcome. Significant correlation of *SMARCA2* gene expression with expression of *PKM2* and *PRKAA1*, genes involved in glucose metabolism, is observed (d). Low *SMARCA2* expression associates with high transcript level of *PKM2* gene, *p*‐value < .001 (e). There is a strong association of *PKM2* transcript level with molecular stage of bladder cancer: neuronal, *p*‐value = 1.99E‐02; basal squamous, *p*‐value = 1.62E‐12; luminal, *p*‐value = 7.71E‐01, luminal infiltrated, *p*‐value = 3.76E‐04; luminal papillary, *p*‐value = 2.58E‐08 (f). Low *SMARCA2* expression associates with higher *PRKAA1* gene expression (g)

As SWI/SNF CRC is involved in sucrose metabolism in yeast,^23^ and in the regulation of nutrient sensing and metabolism control in mammals,^24^ we assessed the potential correlation of *SMARCA2* expression with expression of genes coding for main glucose metabolism enzymes including: *FBP1* (fructose‐1,6‐ bisphospatase)*, GAPDH* (glyceraldehyde 3‐phosphate dehydrogenase), *ENO1* (enolase1)*, LDHA* (lactate dehydrogenase)*, PKM2* (pyruvate kinase M)*, ALDOA, ALDOB, ALDOC* (aldolase A, B and C), and *IDH1* (isocitrate dehydrogenase 1)—a Krebs cycle gene as well as *PRKAA1* gene encoding the AMPKα1 subunit of AMP‐activated Protein Kinase (AMPK), the evolutionarily conserved ancient gauge of metabolism. The statistically significant correlation between expression of *SMARCA2* and *PKM*2 and *PRKAA1* genes were found (Figure 4a). In BC samples with low *SMARCA2* expression, a significantly higher expression of *PKM2* was observed (*p*‐value = .0081) compared with samples with high *SMARCA2* expression (Figure 4b). The exhaustive transcriptomic analysis using TCGA datasets indicated the strongest *PKM2* overexpression in basal squamous and luminal papillary BC subtypes (Figure 4c) which exhibited low *SMARCA2* expression (Figure 1d). Similarly to *PKM2*, the *PRKAA1* gene expression was elevated in BC samples with low *SMARCA2* expression (*p*‐value = .0378; Figure 4d). Collectively, our results indicated that BRM encoded by *SMARCA2* gene may be a negative regulator of metabolic processes dependent on PKM2 and AMPK in BC. The GO classification of genes positively correlating with *SMARCA2* expression revealed the enrichment of various GO classes related to metabolic processes: like nucleic acid metabolism, RNA metabolism, cellular metabolic process, heterocycle metabolic process, nucleobase‐containing compound metabolic process, cellular aromatic compound metabolic process and chromosome organization, chromatin organization, and histone modifications (Supplementary Dataset 1).

### 3.5 T24 and 5637 BC cell lines exhibit different molecular and metabolic alterations

**Figure 5:**
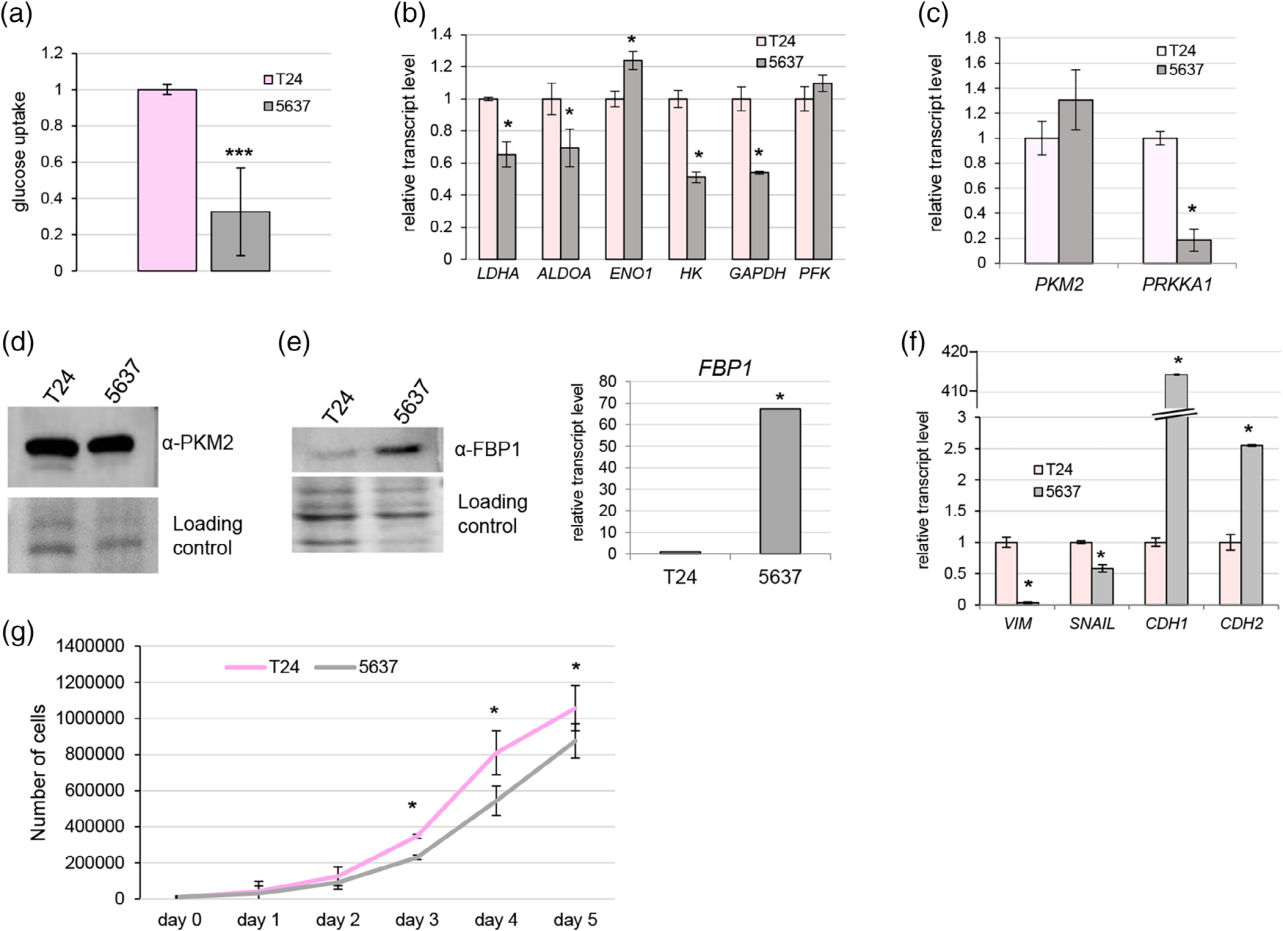
T24 and 5637 bladder cancer cell lines exhibit different glucose metabolisms, EMT markers statuses, and growth rates. The 5637 BC cell line exhibits lower glucose uptake rate compared with T24 cells (a). The expression of glycolysis enzymes encoding genes and *PRKAA1* is significantly lower in 5637 cell line than in T24 (b, c). The PKM2 protein level is slightly higher in T24 bladder cancer cell line than in 5637 line (d). The expression of FBP1 in T24 BC line is lower on both protein and transcript level compared with 5637 cell line (e). The EMT markers *VIM* and *SNAIL* are overexpressed in T24 cells but *CDH1*, an E‐cadherin encoding gene, is dramatically elevated in 5637 cell line (f). T24 cells grow significantly faster than 5637 cells (g). BC, bladder cancer

The T24 and 5637 BC cell lines are commonly used for the molecular study of BC. To verify the glucose metabolism in these two lines the glucose uptake analysis was performed. Interestingly, the T24 cell line exhibited statistically significant stronger glucose uptake than 5637 cell line *p*‐value < .0001 (Figure 5a) suggesting the occurrence of the faster glycolysis flux in T24 cell line. The analysis of expression levels of genes encoding main glycolysis enzymes, namely: *LDHA, ALDOA, ENO1, HK, GAPDH*, and *PFK* indicated higher expression of *LDHA, ALDOA, HK*, and *GAPDH* in T24 compared with 5637 BC cell line (Figure 5b). Interestingly, the expression of AMPKα1 encoding gene was dramatically decreased in 5637 BC cells (Figure 5c). Interestingly, no significant alterations of *PKM2* expression were observed between tested cell lines on both transcript and protein levels (Figure 5c,d). The opposite effect for Fructose‐1,6‐Bisphosphatase (FBP1) enzyme abundance was observed. In T24 line, FBP1 protein level was decreased compared with 5637 cell line (Figure 5e) and consistently the *FBP1* transcript level was more than 60‐fold higher in 5637 BC cells in comparison with T24 cell line (Figure 5e). Moreover, obtained data strongly suggest that these two BC cell lines implement various metabolic programs. T24 and 5637 BC cells also differ phenotypically; the T24 cells are bigger and more spindle‐shaped compared with more compacted 5637 cells (Supplementary Figure 1). The spindle shape resembles more mesenchymal/basal phenotype; therefore, the expression analysis of epithelial to mesenchymal markers (*CDH1, CDH2, SNAIL*, and *VIM*) was performed. As we suspected, in T24 cell lines the level of vimentin and Snail was higher than in 5637 cells (Figure 5f). Interestingly, although the *CDH2*, an N‐cadherin encoding gene, was ~2.5‐fold upregulated in 5637 cell line the level of E‐cadherin encoding gene *CDH1* (the epithelial marker) was more than 400 times higher in 5637 cells (Figure 5f). These results strongly suggest that T24 cell line is more basal than 5637 BC cell line which seems to be more luminal. Additionally, the T24 cell line exhibits statistically significant higher growth rate (Figure 5g) and the T24 cells migrate more collectively than 5637 cells which migrate rather as single cells (Supplementary Figure 2).

### 3.6 T24 and 5637 BC cell lines exhibit differential expression of SWI/SNF CRCs subunits

**Figure 6:**
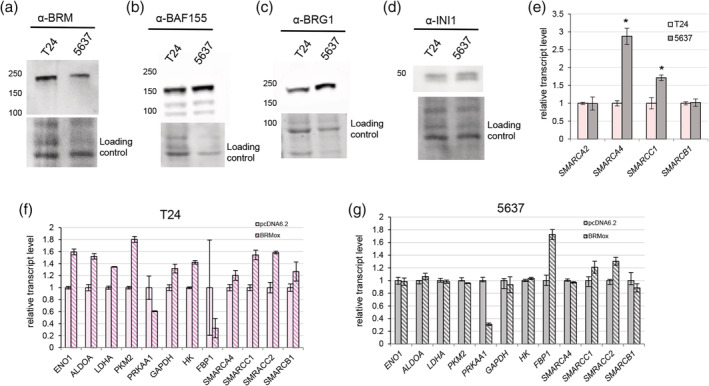
Bladder cancer cell lines exhibit various molecular characteristics. BRM subunit is overexpressed in T24 line compared with 5637 BC cell line (a), BAF155 (b), and BRG‐1 proteins are overaccumulated in 5637 line compared with T24 BC cell line (c). The INI1 protein levels in T24 and 5637 cell lines are similar (d); stain‐free method was used as a loading control in (a), (b), (c), and (d). The genes encoding BRG1 (*SMARCA2*) and BAF155 (*SMARCC1*) subunits of SWI/SNF are expressed differentially in T24 and 5637 cell lines (e). BRM overexpression affects the expression of glucose metabolism genes, *PRKAA1* and SWI/SNF subunits in T24 cell line (f). The overexpression of BRM causes decreased expression of *PRKAA1* gene and elevated level of *FBP1*, *SMARCC1*, and *SMARCC2* genes in 5637 cell line (g). BC, bladder cancer

The SWI/SNF controls the expression of E‐cadherin^25^, ^26^ and is involved in glucose metabolism control; therefore, we evaluated the alterations of SWI/SNF subunits expression in T24 and 5637 BC cell lines. Slight differences between BRM level in T24 and 5637 BC cell lines were detected (Figure 6a). More pronounced expression changes were observed for BAF155 and BRG1 subunits of SWI/SNF CRCs as both proteins were overexpressed in 5637 line compared with T24 cells (Figure 6b,c). The level of INI1, in both lines was comparable (Figure 6d). The specificity of used antibody against BAF155 and BRM was validated on breast cancer cell line with amiRNA targeting *SMARCC1* or *SMARCA2* (Supplementary Figure 4). The INI1 antibody used in both IHC and WB analysis was validated in Sarnowska et al.^10^ The evaluation of transcript level indicated that *SMARCA4* (BRG1) and *SMARCC1* (BAF155) consistently exhibited elevated expression in 5637 cell line, while the expression level of *SMARCA2* (BRM) was comparable in both cell lines (Figure 6e). *PKM* and *PRKAA1* expression correlates with *SMARCA2*; SWI/SNF subunits encoding genes are regulated by themselves^10^; and SWI/SNF CRCs are involved in control of glucose metabolism genes; therefore, we investigated the influence of BRM overexpression on the expression on glycolysis enzymes encoding genes, SWI/SNF subunits and *PRKAA1*. The transient overexpression of BRM was performed in both T24 and 5637 cell lines, respectively (Supplementary Figure 5). Interestingly, the BRM overexpression caused elevated expression of almost all examined glycolysis genes including *PKM2* except *FBP1* in T24 BC cell lines, but not in 5637 cells, where *FBP1* increased after BRM overexpression. By contrast, the *PRKAA1* transcript was downregulated in both T24 and 5637 cell lines (Figure 6f,g). Similarly, in T24 line BRM induced the higher expression of all examined SWI/SNF encoding genes, but in 5637 only *SMARCC1*/BAF155 and *SMARCC2*/BAF170 increased while *SMARCA4* and *SMARCB1* expression levels remained unaltered (Figure 6f,g). This observation suggested the different roles of BRM and BRM containing SWI/SNF CRC in these two lines.

### 3.7 Low expression of FBP1 gene is related to patient poor outcome

**Figure 7:**
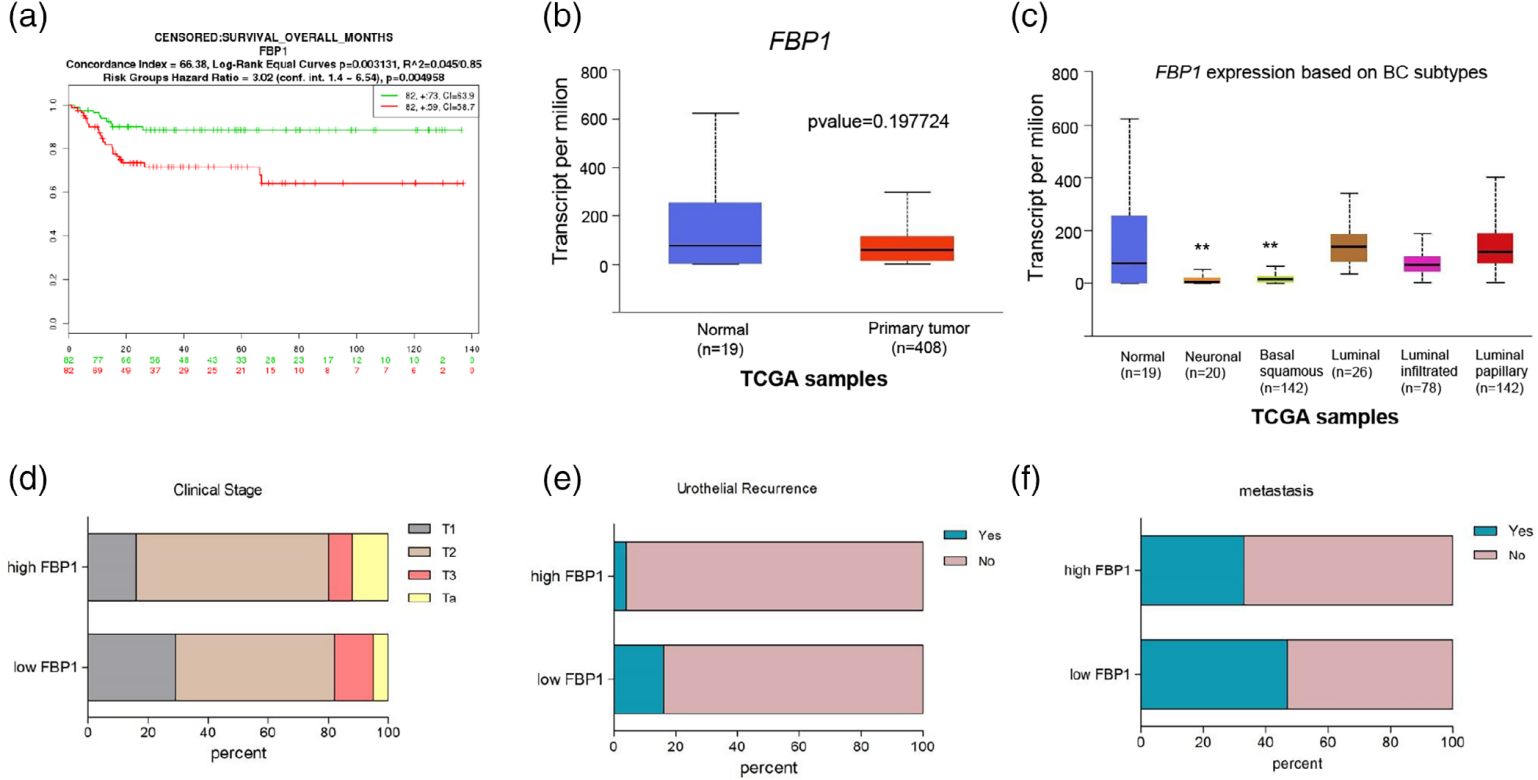
Impairment of *FBP1* affects the BC patients' outcome. *FBP1* expression is associated with BC patients' survival (a). *FBP1* gene is expressed at similar levels in primary BC tumor and healthy tissue, *p*‐value = .197724 (b). *FBP1* is expressed differentially in various BC molecular subtypes: neuronal, *p*‐value = 8.14E‐03; basal squamous, *p*‐value = 9.49E‐03; luminal, *p*‐value = 9.81E‐01, luminal infiltrated, *p*‐value = 1.86E‐01, and luminal papillary, *p*‐value = 8.19E‐01 (c). *FBP1* gene expression is significantly associated with clinical stage of the disease, *p*‐value = .0312 (d), urothelial recurrence *p*‐value = .0047 (e), and metastasis, *p*‐value = .030 (f). BC, bladder cancer

To assess the impact of *FBP1* expression on BC patients' survival the analysis of GEO database accession number GSE13507 was performed using SurvExpress approach. The low expression of *FBP1* strongly correlated with poor survival of BC patients (Figure 7a). Interestingly, the poor survival was independent on muscle invasion. The TCGA datasets analysis indicated no differences in *FBP1* expression in BC cells compared with healthy tissue (Figure 7b), although the strong downregulation was observed in neuronal (*p*‐value = 8.14E‐03) and basal squamous (*p*‐value = 9.49E‐03) BC subtypes (Figure 7c). Moreover, the analysis of GSE31684 datasets indicated that the low *FBP1* expression level correlates with clinical stage of the disease (Figure 7d), more frequent urothelial recurrence (Figure 7e) and metastasis (Figure 7f). Thus, the *FBP1* expression may serve as a prognostic marker for BC patients.

## 4 DISCUSSION

In 2014, 262 MIBC samples were analyzed, and the two main basal and luminal BC subtypes were identified as a result of this study. These molecular subtypes resembled the breast cancer, where such subtypes are also defined.^6^ More deep analysis revealed that BC may be divided to more molecular subtypes. Currently, based on TCGA data from 2017 in MIBC five subtypes were identified, namely luminal‐papillary, luminal‐infiltrated, luminal, basal squamous, and neuronal.^27^ In basal subtypes of breast cancer—triple negative breast cancer,^28^, ^29^ the growth and proliferation depend on the BRM and BRG1 central ATPases of the SWI/SNF CRCs, which is involved in the control of numerous regulatory processes.^30^ In samples of advanced MIBC the strong decrease of BRM protein level corresponding to downregulation of *SMARCA2* gene expression was detected. Further analysis indicated that the impairment of SWI/SNF CRCs in BC is not restricted to the BRM ATPase subunit only, as INI1 and BAF155, another SWI/SNF core subunits were less abundant in cancer cells. However, *SMARCB1* gene encoding INI1 protein was strongly upregulated suggesting affected regulation at the posttranscriptional and/or protein level in BC. Although, in some particular cases the levels of SWI/SNF subunits were intact in cancer cells compared with healthy urothelium strongly suggesting the BC heterogeneity in terms of SWI/SNF complex abundance.

The *SMARCA2* expression was consistently downregulated in BC and its expression strongly correlated with metastasis. The patients with low *SMARCA2* expression exhibited two times more frequent metastasis rate than patients with high *SMARCA2* transcript level. Additionally, patients with low *SMARCA2* expression had lower recurrence free survival and overall survival. Interestingly, this observation was confirmed only for MIBC, what strongly indicated that the impairment of BRM and SWI/SNF complex may be characteristic for higher aggressive BC.

SWI/SNF complex is known as sucrose metabolism regulator in yeast. In human cells, SWI/SNF CRCs control lipid metabolism,^31^ nutrient metabolism, and signaling.^24^ Therefore, we assessed the association of *SMARCA2* expression with the main glucose metabolism genes. Surprisingly, the *SMARCA2* correlated with *PKM2* gene expression, which encodes a pyruvate kinase M and expression of *PRKAA1* gene coding for the AMPKα1 subunit of AMPK, which controls metabolic homeostasis in the cells.^32^ Inactivation of AMPK leads to metabolic imbalance in the cell and promotes metabolic reprogramming. In BC, the low expression of *SMARCA2* correlates with higher *PRKAA1* and *PKM2* expression indicative of its important role in the control of metabolic processes in this cancer type. The increased PKM2 level was observed in BC cell resistant to cisplatin.^33^ Additionally, the TCGA BLCA database analysis indicated the *SMARCA2* correlation with expression of genes involved in chromatin organization, transcription and various metabolic processes including nucleic acid metabolic processes, RNA metabolism, cellular metabolic processes, etc. (Supplementary Dataset 1). Thus, given the observed alteration in the control of metabolism, our study opens a new attractive, so far unexplored path which may lead to better characterization of this disease.

Interestingly, during our study we found that the BC T24 and 5637 cell lines exhibit differential alterations in the SWI/SNF subunit abundance as well as in the degree of metabolism‐related deregulations including glucose uptake and expression of glycolysis enzymes encoding genes. Further, in T24 BC cell line the vimentin and Snail, the EMT markers were upregulated, as well as E‐cadherin, supporting the hypothesis that T24 and 5637 cell lines represent two different molecular subtypes of BC. T24 is more basal‐like but 5637 is more luminal BC cell line. The T24 cell line exhibits higher growth rate what is characteristic for more aggressive basal subtype of BC. This finding, together with the observation that the different BC molecular subtypes are featured by various extents of SWI/SNF impairment as well as expression levels of metabolic enzymes, lead us to the conclusion that the alterations of SWI/SNF complex may be a signature for these cancer subtypes. Molecularly, BC subtypes resemble the breast cancer and both basal‐like breast cancer^34^ and basal squamous BC exhibits dramatic downregulation of *FBP1* gene which controls glycolysis flux and gluconeogenesis. In breast cancer, low or absent expression of *FBP1* gene was associated with reduced Disease‐Free Survival.^35^ Consistently, we observed the correlation of downregulation of *FBP1* expression with reduced overall survival. The loss of FBP1 in basal‐like breast cancer cells causes increased glucose uptake and macromolecule biosynthesis, increased PKM2 activity, maintenance of ATP production under hypoxic condition and is an essential oncogenic event in epithelial to mesenchymal transition of cancer cells.^34^ In T24 BC cell line, the low expression of FBP1 was observed and higher glucose uptake compared with 5637 cell line, further indicating that T24 BC cell line resembles basal‐like BC subtype.

Here we provided the first evidences that the impairment of chromatin remodeling machinery, the SWI/SNF complex, which precisely controls gene expression has a great importance in the development, progression and molecular characteristics of BC subtypes. Given the observed differences between molecular subtypes and MIBC versus NMIBC we propose the SWI/SNF BRM subunit as a biomarker for some BC types. Our results indicate that the current study of BC biology should be directed toward the better molecular characteristics of metabolic and transcriptomic events in this cancer type which may lead to the identification of a solid basis for development of new BC treatment strategies.

## 5 CONCLUSIONS

Summarizing, significant downregulation of SWI/SNF core BRM, INI1, and BAF155 subunits was found in MIBC. Low expression of *SMARCA2*, a BRM encoding gene, associates with metastasis, reduced survival for MIBC patients and correlates with *PKM2* and *PRKAA1* genes expression. Moreover, low *FBP1* expression is associated with poor survival for patients, local recurrence, and metastasis.

## CONFLICT OF INTEREST

No conflict of interest exits and manuscript has been approved by all authors for publication.

## AUTHOR CONTRIBUTIONS

M.S., M.Sz., A.O., I.J., and N.R. performed experiments and M.S. additionally wrote part of the manuscript; M.Sz. and A.C. selected and verified bladder cancer samples; J.A.S. and T.D. analyzed the data; T.J.S. and E.S. designed experiments, analyzed data, and wrote manuscript.

## Supporting information


**Supplementary Figure 1** The T24 and 5637 BC exhibit various phenotypes, magnification 63x.
Supplementary Figure 2. T24 cell line migrate differentially than 5637 cell line in scratch assay analysis.
Supplementary Figure 3. Transient overexpression of BRM in T24 and 5637 cell lines comparing to mock transfection using empty vector.
Supplementary Figure 4. Anti‐BAF155 and anti‐BRM antibody validation on knockdown BAF155 and BRM cell lines produced using amiRNA system.

**Supplementary Table 1** Catalogue of monoclonal antibodies used in immunohistochemistry.
Supplementary Table 2. Primers used for qRT‐PCR analysis.
Supplementary Table 3. Antibodies used in western blot analysis.

**Supplementary Dataset 1** Gene Ontology classification of genes positively correlating with SMARCA2 expression in BLCA
