# Supplementary material for: SWI/SNF chromatin remodeling complex and glucose metabolism are deregulated in advanced bladder cancer
Source: IUBMB Life. 2020 Feb 19;72(6):1175–88. doi: 10.1002/iub.2254 (PMC7317882; doi:10.1002/iub.2254)
Supplement: Supplementary file 1 — Supplementary Figure 1 The T24 and 5637 BC exhibit various phenotypes, magnification 63x. Supplementary Figure 2. T24 cell line migrate differentially than 5637 cell line in scratch assay analysis. Supplementary Figure 3. Transient overexpression of BRM in T24 and 5637 cell lines comparing to mock transfection using empty vector. Supplementary Figure 4. Anti‐BAF155 and anti‐BRM antibody validation on knockdown BAF155 and BRM cell lines produced using amiRNA system. [file IUB-72-1175-s001.docx]

**Supplementary material**

**
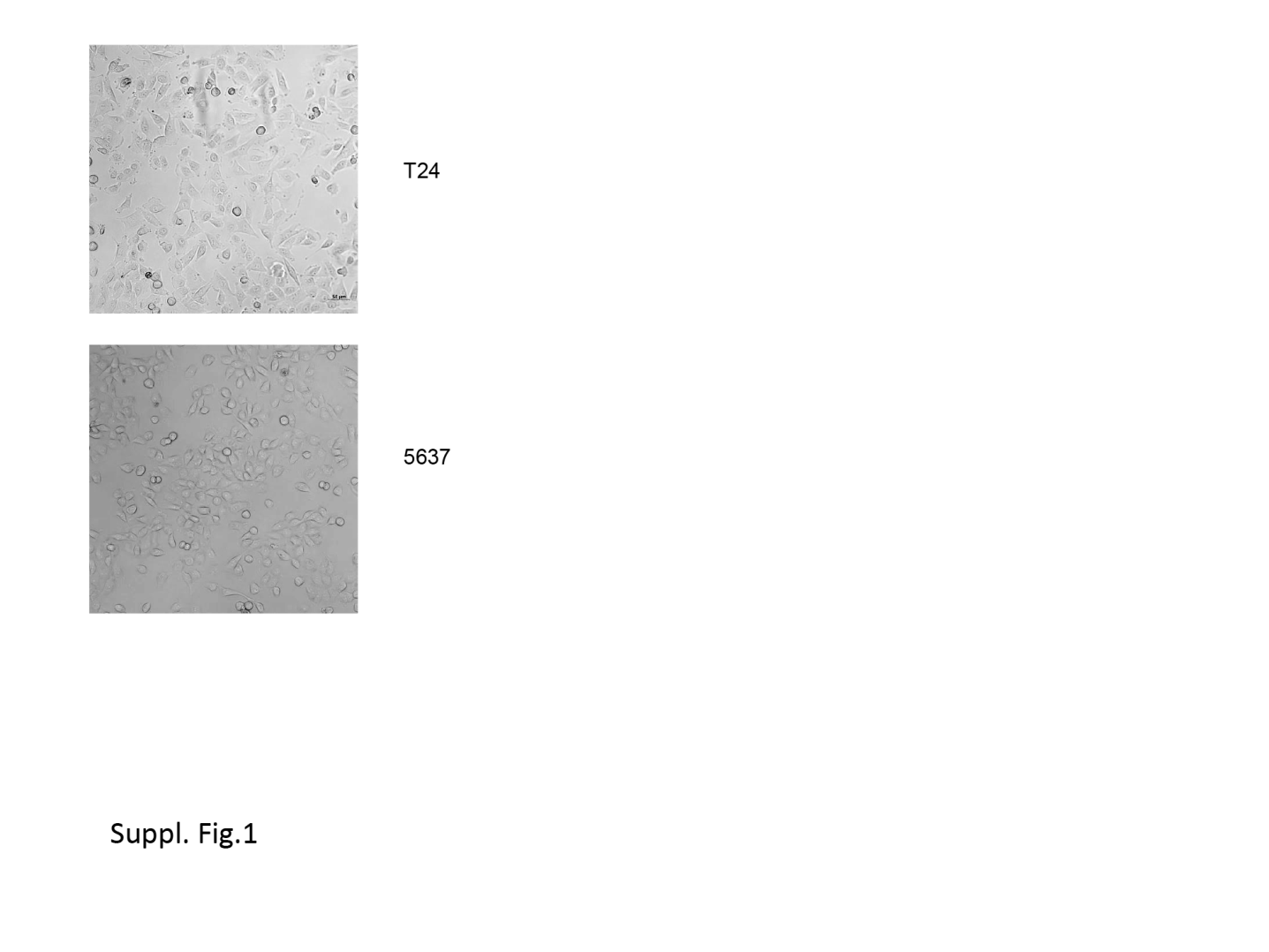
**

Supplementary Figure1. The T24 and 5637 BC exhibit various phenotypes, magnification 63x.


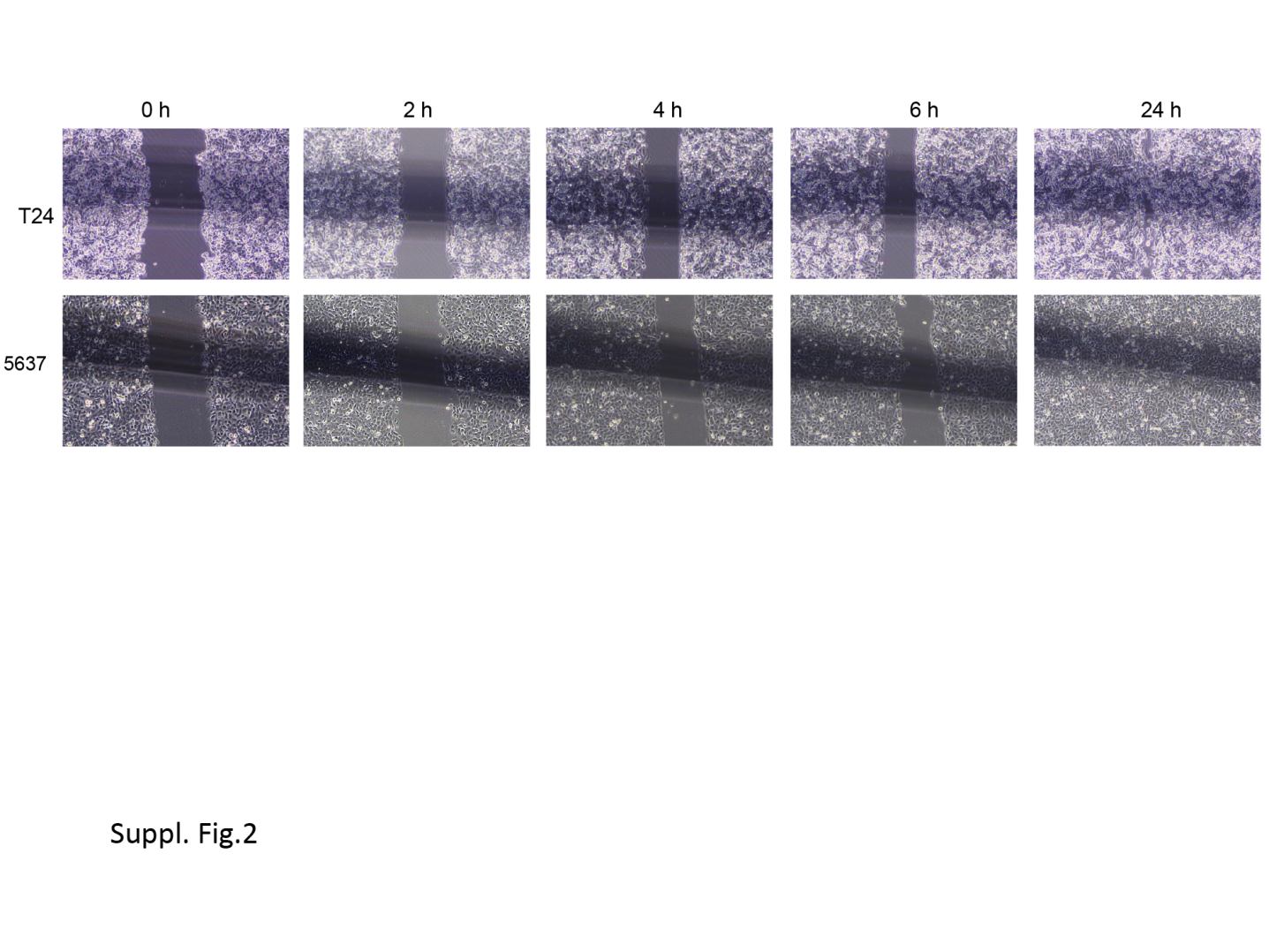


Supplementary Figure 2. T24 cell line migrate differentially than 5637 cell line in scratch assay analysis.


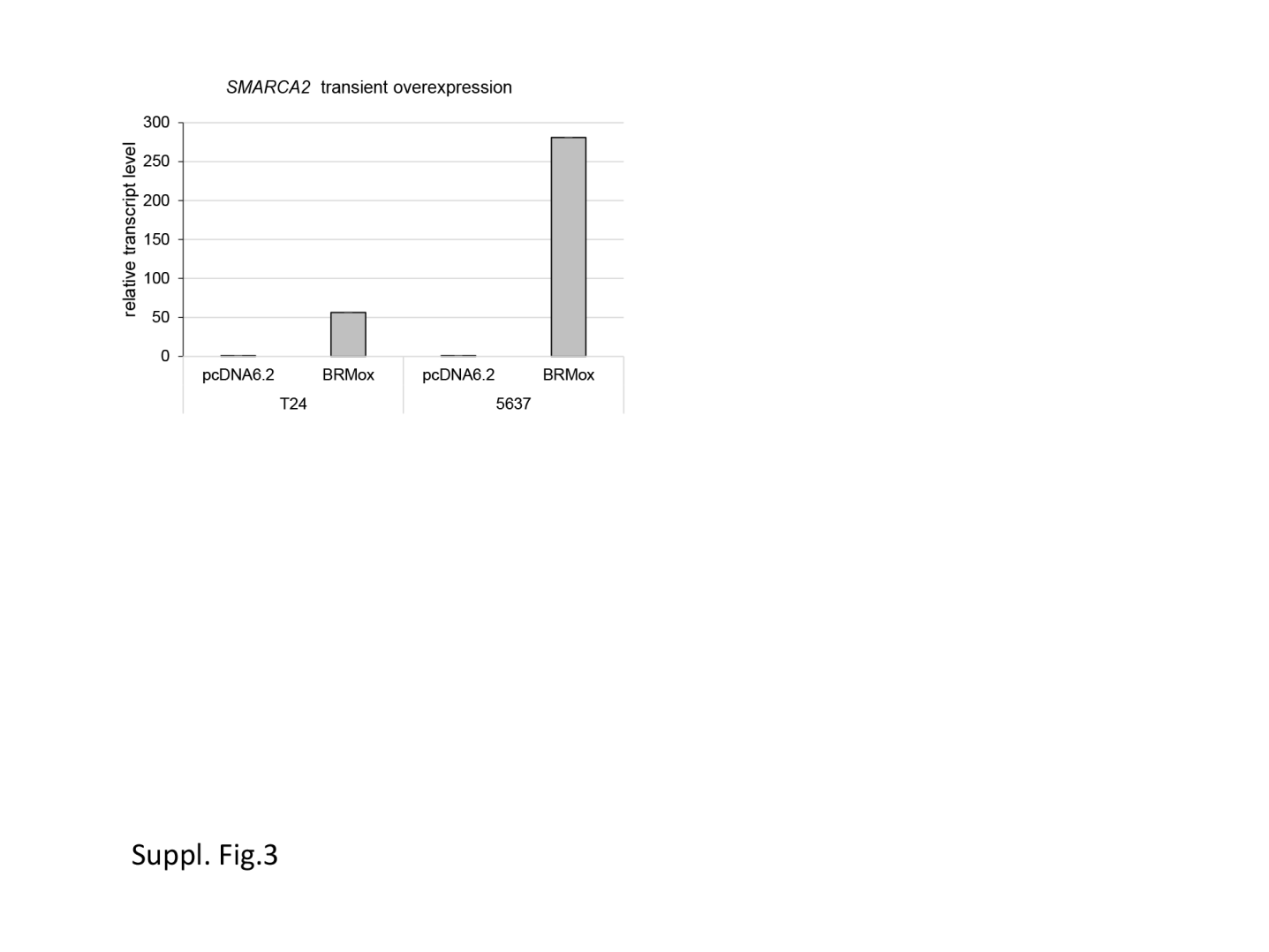


Supplementary Figure 3. Transient overexpression of BRM in T24 and 5637 cell lines comparing to mock transfection using empty vector.


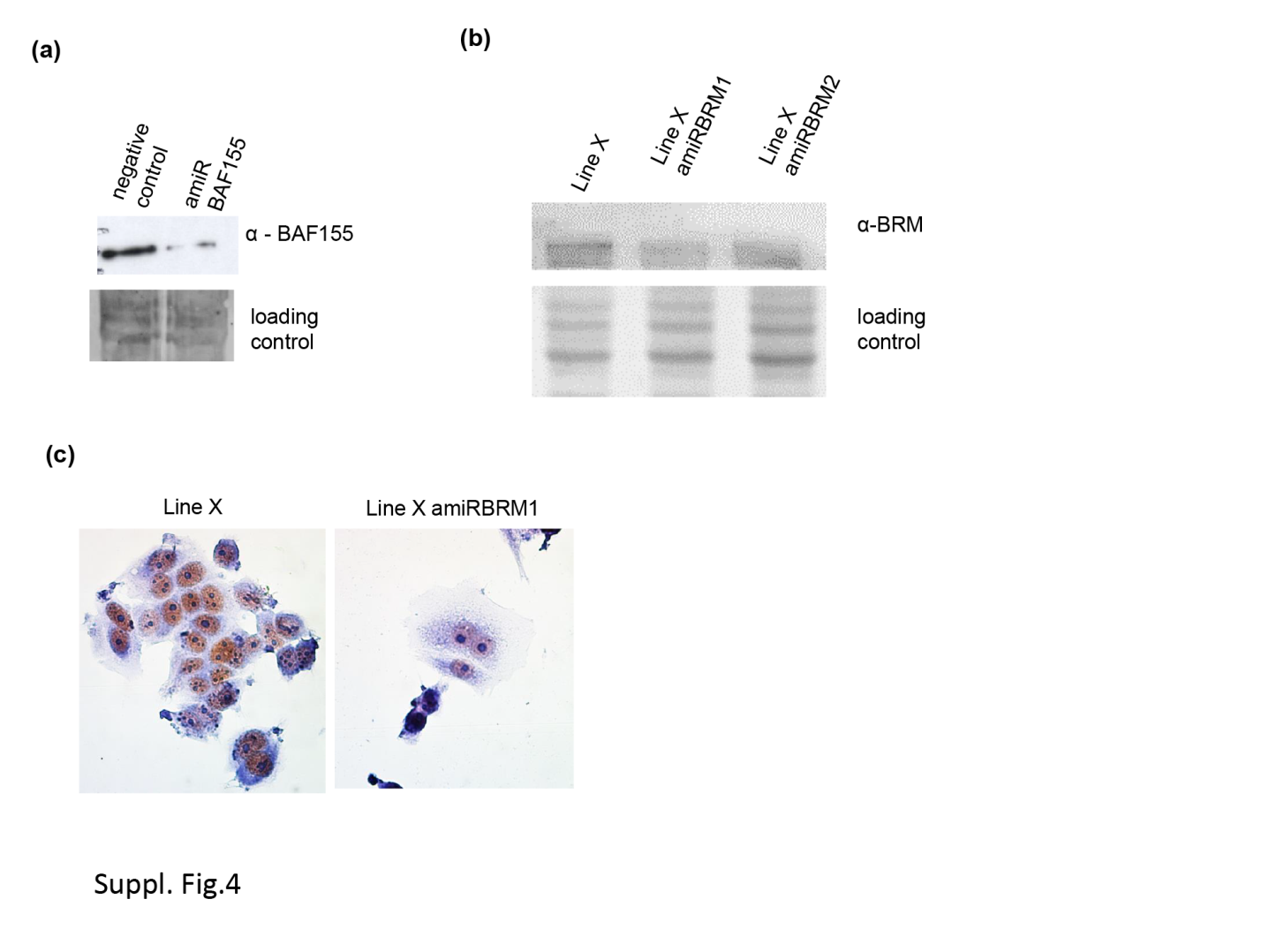


Supplementary Figure 4. Anti-BAF155 and anti-BRM antibody validation on knockdown BAF155 and BRM cell lines produced using amiRNA system.

**Supplementary dataset 1.** Gene Ontology classification of genes positively correlating with SMARCA2 expression in BLCA

**Supplementary Table 1. Catalogue of monoclonal antibodies used in immunohistochemistry**

| **Antibody** | **Cat. No.** | **Concentration** | **Incubation conditions** |
| --- | --- | --- | --- |
| **BRM (D9E8B)** | CST, #11966 | 1:200 | 1 h, RT |
| **INI1 (D9C2)** | CST, #8745 | 1:200 | 1 h, RT |
| **BAF155 (D7F8S)** | CST, #11956 | 1:200 | 1 h, RT |

**Supplementary Table 2. Primers used for qRT-PCR analysis.**

| **Gene** | **Sequence** |
| --- | --- |
| ***SMARCA2* (BRM)** | F: CGGTTTGATTGTGCCTGGTT |
|  | R: GCTTTTGTTCAGATCATAGAGCAT |
| ***SMARCA4* (BRG1)** | F: GACATTCCAGTCTCGACCCC |
|  | R: GCAACAGTACTGCCAGCAAC |
| ***SMARCC1* (BAF155)** | F: GCCTGGCTTTCTCACTTCAC |
|  | R: CTGAGGGTTTGAAAGGCAAA |
| ***SMARCB1* (INI1)** | F: GACCAGGACAGGAACACGAG |
|  | R: CAAATGGAATGTGTGCCGG |
| ***FBP1*** | F: TGACCCAGCTGCTCAACTC |
|  | R: TGATCACCTGTCACGTTGGT |
| ***PKM2*** | F: GTCTGAATGAAGGCAGTCCC |
|  | R: TGCAGTGGAGCTCAGAGAGA |
| ***PRKAA2* (AMPK)** | F: TCTCCCTATCACTATCAGAAGTTG |
|  | R: AGGTTACGTGACTTGCTGCT |
| ***UBC*** | F: ATTTGGGTCGCGGTTCTTG |
|  | R: TGCCTTGACATTCTCGATGGT |
| ***LDHA*** | F: GCCAGAGACAATCTTTGGTG |
|  | R: GGCCTGTGCCATCAGTATCT |
| ***ALDOA*** | F: GACTCATCTGCAGCCAGGA |
|  | R: GAATTTCCTCTGAAGCACGC |
| ***ENO1*** | F: GCCTCCTGCTCAAAGTCAAC |
|  | R: AACGATGAGACACCATGACG |
| ***PFK*** | F: ATCATGACCCATGAAGAGCA |
|  | R: CCCTGACAGCAGCATTCATA |
| ***HK1*** | F: GAACTGGACCGTCTGAATGT |
|  | R: ACAGTTCCTTCACCGTCTGG |
| ***GAPDH*** | F: GAAGGTGAAGGTCGGAGTC |
|  | R: GAAGATGGTGATGGGATTTC |
| ***CDH1*** | F: AGGCCAAGCAGCAGTACATT |
|  | R: AAATGTGTCTGGCTCCTGGG |
| ***CDH2*** | F: CCTTTCAAACACAGCCACGG |
|  | R: TGTTTGGGTCGGTCTGGATG |
| ***SNAIL*** | F: AATCCAGAGTTTACCTTCCAG |
|  | R: CAGAGTCCCAGATGAGCATT |
| ***VIM*** | F: CAATGTTAAGATGGCCCTTG |
|  | R: GGGTATCAACCAGAGGGAGT |

**Supplementary Table 3. Antibodies used in western blot analysis.**

| **Antibody** | **Cat. No.** | **Primary Ab concentration** | **Incubation buffer** | **Host** | **Secondary Ab concentration** |
| --- | --- | --- | --- | --- | --- |
| **BRM (D9E8B)** | CST, #11966 | 1:1000 | 5% milk in TBS-T | rabbit | 1:10000 |
| **BRG1 (D1Q7F)** | CST, #49360 | 1:1000 | 5% BSA in TBS-T | rabbit | 1:10000 |
| **INI1 (D9C2)** | CST, #8745 | 1:1000 | 5% milk in TBS-T | rabbit | 1:10000 |
| **BAF155 (D7F8S)** | CST, #11956 | 1:1000 | 5% milk in TBS-T | rabbit | 1:10000 |
| **FBP1 (EPR4619)** | Abcam, ab109020 | 1:1000 | 5% milk in TBS-T | rabbit | 1:10000 |
| **PKM2** | CST, #3198 | 1:1000 | 5% milk in TBS-T | rabbit | 1:10000 |
