# Supplementary material for: SWI/SNF chromatin remodeling complex and glucose metabolism are deregulated in advanced bladder cancer
Source: IUBMB Life. 2020 Feb 19;72(6):1175–88. doi: 10.1002/iub.2254 (PMC7317882; doi:10.1002/iub.2254)
Supplement: Supplementary file 2 — Supplementary Table 1 Catalogue of monoclonal antibodies used in immunohistochemistry. Supplementary Table 2. Primers used for qRT‐PCR analysis. Supplementary Table 3. Antibodies used in western blot analysis. [file IUB-72-1175-s002.docx]

**Supplementary Table 1. Catalogue of monoclonal antibodies used in immunohistochemistry**

| **Antibody** | **Cat. No.** | **Concentration** | **Incubation conditions** |
| --- | --- | --- | --- |
| **BRM (D9E8B)** | CST, #11966 | 1:200 | 1 h, RT |
| **INI1 (D9C2)** | CST, #8745 | 1:200 | 1 h, RT |
| **BAF155 (D7F8S)** | CST, #11956 | 1:200 | 1 h, RT |

**Supplementary Table 2. Primers used for qRT-PCR analysis.**

| **Gene** | **Sequence** |
| --- | --- |
| ***SMARCA2* (BRM)** | F: CGGTTTGATTGTGCCTGGTT |
|  | R: GCTTTTGTTCAGATCATAGAGCAT |
| ***SMARCA4* (BRG1)** | F: GACATTCCAGTCTCGACCCC |
|  | R: GCAACAGTACTGCCAGCAAC |
| ***SMARCC1* (BAF155)** | F: GCCTGGCTTTCTCACTTCAC |
|  | R: CTGAGGGTTTGAAAGGCAAA |
| ***SMARCB1* (INI1)** | F: GACCAGGACAGGAACACGAG |
|  | R: CAAATGGAATGTGTGCCGG |
| ***FBP1*** | F: TGACCCAGCTGCTCAACTC |
|  | R: TGATCACCTGTCACGTTGGT |
| ***PKM2*** | F: GTCTGAATGAAGGCAGTCCC |
|  | R: TGCAGTGGAGCTCAGAGAGA |
| ***PRKAA2* (AMPK)** | F: TCTCCCTATCACTATCAGAAGTTG |
|  | R: AGGTTACGTGACTTGCTGCT |
| ***UBC*** | F: ATTTGGGTCGCGGTTCTTG |
|  | R: TGCCTTGACATTCTCGATGGT |
| ***LDHA*** | F: GCCAGAGACAATCTTTGGTG |
|  | R: GGCCTGTGCCATCAGTATCT |
| ***ALDOA*** | F: GACTCATCTGCAGCCAGGA |
|  | R: GAATTTCCTCTGAAGCACGC |
| ***ENO1*** | F: GCCTCCTGCTCAAAGTCAAC |
|  | R: AACGATGAGACACCATGACG |
| ***PFK*** | F: ATCATGACCCATGAAGAGCA |
|  | R: CCCTGACAGCAGCATTCATA |
| ***HK1*** | F: GAACTGGACCGTCTGAATGT |
|  | R: ACAGTTCCTTCACCGTCTGG |
| ***GAPDH*** | F: GAAGGTGAAGGTCGGAGTC |
|  | R: GAAGATGGTGATGGGATTTC |
| ***CDH1*** | F: AGGCCAAGCAGCAGTACATT |
|  | R: AAATGTGTCTGGCTCCTGGG |
| ***CDH2*** | F: CCTTTCAAACACAGCCACGG |
|  | R: TGTTTGGGTCGGTCTGGATG |
| ***SNAIL*** | F: AATCCAGAGTTTACCTTCCAG |
|  | R: CAGAGTCCCAGATGAGCATT |
| ***VIM*** | F: CAATGTTAAGATGGCCCTTG |
|  | R: GGGTATCAACCAGAGGGAGT |

**Supplementary Table 3. Antibodies used in western blot analysis.**

| **Antibody** | **Cat. No.** | **Primary Ab concentration** | **Incubation buffer** | **Host** | **Secondary Ab concentration** |
| --- | --- | --- | --- | --- | --- |
| **BRM (D9E8B)** | CST, #11966 | 1:1000 | 5% milk in TBS-T | rabbit | 1:10000 |
| **BRG1 (D1Q7F)** | CST, #49360 | 1:1000 | 5% BSA in TBS-T | rabbit | 1:10000 |
| **INI1 (D9C2)** | CST, #8745 | 1:1000 | 5% milk in TBS-T | rabbit | 1:10000 |
| **BAF155 (D7F8S)** | CST, #11956 | 1:1000 | 5% milk in TBS-T | rabbit | 1:10000 |
| **FBP1 (EPR4619)** | Abcam, ab109020 | 1:1000 | 5% milk in TBS-T | rabbit | 1:10000 |
| **PKM2** | CST, #3198 | 1:1000 | 5% milk in TBS-T | rabbit | 1:10000 |
